# Signs of immunosenescence correlate with poor outcome of mRNA COVID-19 vaccination in older adults

**DOI:** 10.1038/s43587-022-00292-y

**Published:** 2022-10-14

**Authors:** Miguel Ángel Palacios-Pedrero, Janina M. Jansen, Cornelia Blume, Nils Stanislawski, Rebecca Jonczyk, Antonia Molle, Mariana Gonzalez Hernandez, Franziska K. Kaiser, Klaus Jung, Albert D. M. E. Osterhaus, Guus F. Rimmelzwaan, Giulietta Saletti

**Affiliations:** 1 Research Center for Emerging Infections and Zoonoses, University of Veterinary Medicine, Hanover, Germany; 2 Institute of Technical Chemistry, Leibniz University, Hanover, Germany; 3 Institute of Microelectronic Systems, Leibniz University, Hanover, Germany; 4 Institute for Animal Breeding and Genetics, Genomics and Bioinformatics of Infectious Diseases, University of Veterinary Medicine, Hanover, Germany; 5 Global Virus Network, Center of Excellence, Buffalo, NY USA

**Keywords:** Infection, Infectious diseases, Ageing

## Abstract

Vaccination against severe acute respiratory syndrome coronavirus 2 (SARS-CoV-2) is effective in preventing COVID-19 hospitalization and fatal outcome. However, several studies indicated that there is reduced vaccine effectiveness among older individuals, which is correlated with their general health status^1,2^. How and to what extent age-related immunological defects are responsible for the suboptimal vaccine responses observed in older individuals receiving SARS-CoV-2 messenger RNA vaccine, is unclear and not fully investigated^1,3–5^. In this observational study, we investigated adaptive immune responses in adults of various ages (22–99 years old) receiving 2 doses of the BNT162b2 mRNA vaccine. Vaccine-induced Spike-specific antibody, and T and memory B cell responses decreased with increasing age. These responses positively correlated with the percentages of peripheral naïve CD4^+^ and CD8^+^ T cells and negatively with CD8^+^ T cells expressing signs of immunosenescence. Older adults displayed a preferred T cell response to the S2 region of the Spike protein, which is relatively conserved and a target for cross-reactive T cells induced by human ‘common cold’ coronaviruses. Memory T cell responses to influenza virus were not affected by age-related changes, nor the SARS-CoV-2-specific response induced by infection. Collectively, we identified signs of immunosenescence correlating with the outcome of vaccination against a new viral antigen to which older adults are immunologically naïve. This knowledge is important for the management of COVID-19 infections in older adults.

## Abstract (web summary)

COVID-19 vaccines protect against infection, hospitalization and death in older adults, but their effectiveness is lower in this age group compared to young adults. Here, Palacios-Pedrero et al. show that age-dependent signs of immunosenescence in B and T cells in older adults correlate with poor immunological outcomes after mRNA COVID-19 vaccination but not after natural SARS-CoV-2 infection.

## Main

**Fig. 1:**
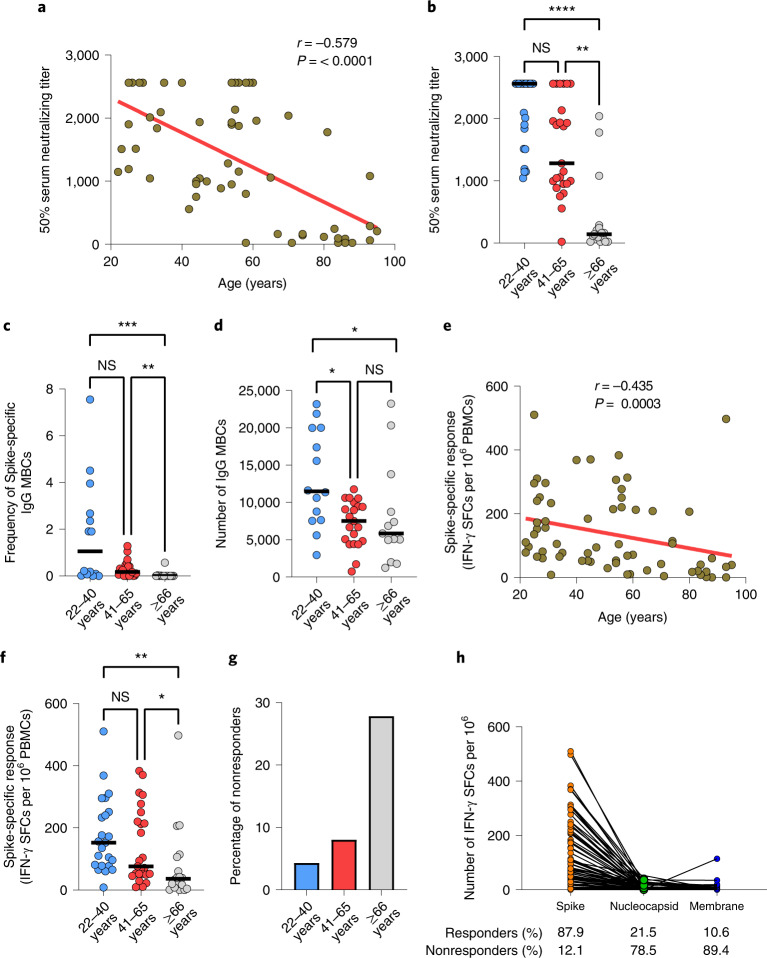
Age-dependent reduction of SARS-CoV-2 neutralizing antibodies, Spike-specific IgG MBCs and T cells in vaccinated individuals. **a**, Correlation between age of study subjects and serum virus-neutralizing antibody titer (*n* = 66).﻿ **b**, Serum virus-neutralizing antibody titers in different age groups (light blue, 22–40 years, *n* = 23; red, 41–65 years, *n* = 25; gray, ≥66 years: *n* = 17). **c**,**d**, Frequency of Spike-specific IgG MBCs (**c**) and number of total IgG MBCs per million of in vitro-expanded PBMCs (**d**) (light blue, 22–40 years, *n* = 14; red, 41–65 years, *n* = 21; gray, ≥66 years, *n* = 13). **e**, Correlation between the age of the study participants and frequency of Spike-specific IFN-γ-secreting T cells (*n* = 66). **f**, Frequency of IFN-γ SFCs after stimulation with Spike in different age groups (light blue, 22–40 years, *n* = 23; red, 41–65 years, *n* = 25; gray, ≥66 years, *n* = 18). **g**, Percentage of individuals with Spike-specific response below the cutoff and considered nonresponders. **h**, Frequency of IFN-γ SFCs in response to nucleocapsid (65 out of 66), membrane (66 out of 66) and Spike (66 out of 66) proteins of SARS-CoV-2 in unexposed vaccinated individuals (*n* = 66). The numbers below the graph represent the percentage of responders and nonresponders for each tested antigen (orange, Spike; green, nucleocapsid; blue, membrane). Each dot represents a single study participant and the horizontal lines indicate the medians. The cutoff value for a positive response is defined as described in the Methods. The red line represents the linear regression; a two-tailed Spearman test was used to test the significance (*r* and *P* values). *P* values for age groups comparison were determined by two-tailed Kruskal–Wallis test with Dunn’s multiple comparison correction; *P < 0.05; **P < 0.01; ***P < 0.001; ****P < 0.0001; NS, not significant. Source data

**Extended Data Fig. 1:**
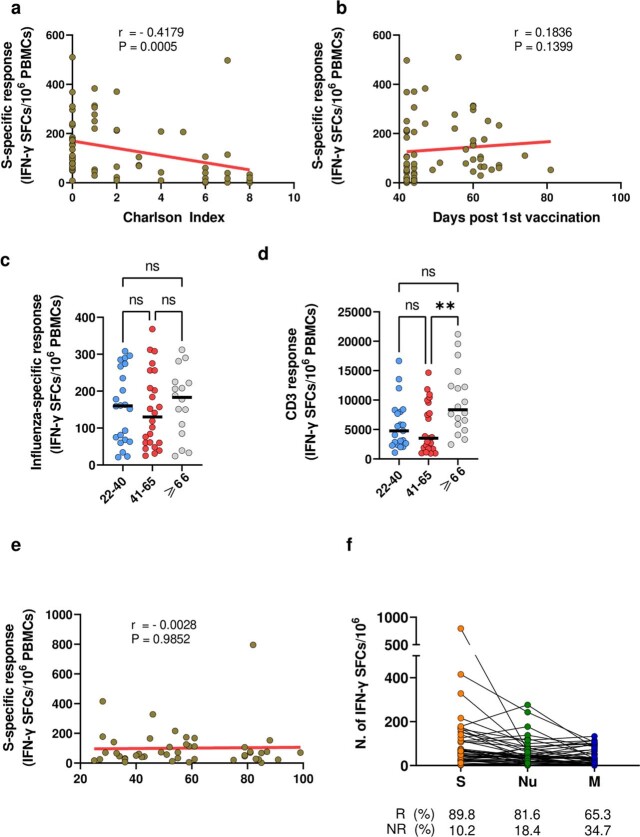
Controls and correlations between spike-specific responses and Charlson Index, days post vaccination or age. (a) Correlation between frequency of S-specific IFN-γ response and Charlson co-morbidities index (r = −0.417, P = 0.0005) or (b) days after the first vaccination (not significant). (c) Influenza (light blue, n = 22; red n = 24; grey n = 16) and (d) CD3 (light blue, n = 22; red n = 23; grey n = 18) specific response in vaccinated subjects of various ages. (e) S-specific IFN-γ response in SARS-CoV-2 exposed subjects (n = 46) and correlation with age (not significant). (f) Frequency of IFN-γ SFCs, in response to Spike (S; orange), Nucleocapsid (Nu; green), and Membrane (M; blue) proteins in SARS-CoV-2 exposed subjects (n = 49). The numbers in the below the graph represent the percentage of responders (R) and non-responders (NR) for each antigen. The cut-off value for a positive response is defined as described in the Methods section. Each dot represents a single study subject and horizontal lines indicate medians. The red line represents linear regression and two-tailed Spearman’s test was used to the test the significance (r and P values). P values were determined by two-tailed Kruskal-Wallis test with Dunn’s multiple comparison correction; ** P < 0.01; ns: not significant; yr: years. Source data

Sixty-six individuals (median age 54; range 22–95) with no history of SARS-CoV-2 infection or related symptoms (hereafter ‘unexposed’) were recruited for this study and blood was drawn between 42 and 81 d (median 44 d) post-first vaccination. PCR-confirmed SARS-CoV-2 infected individuals (hereafter ‘exposed’) (*n* = 49; median age 54 years; range 22–99) were also included (Supplementary Tables 1 and 2)^6^. Although minimal correlates of protection to COVID-19 have not been established so far, vaccine-induced virus-neutralizing antibodies have been implied in protection against infection^7^. Therefore, we first measured SARS-CoV-2 neutralizing antibodies (VN) in our study participants. As previously reported, we found an age-dependent decrease of vaccine-induced neutralizing antibodies (*r* = −0.579; *P* < 0.0001) with significantly lower titers in older adults (≥66 years) compared to young (22–40 years; *P* < 0.0001) and middle-aged (41–65 years; *P* < 0.01) individuals (Fig. 1a,b). A similar correlation was found with the frequency of Spike-specific IgG memory B cells (MBCs) and total number of IgG MBCs, measured in a subset of individuals (22–40, 14 out of 23; 41–65, 21 out of 25; ≥66, 13 out of 18) (Fig. 1c,d). We could not detect Spike-specific IgA MBCs, perhaps because of their low frequency in peripheral blood below the detection limit of our assay. Overall, we observed reduced induction of SARS-CoV-2-specific antibodies and MBCs in older adults, as the possible consequence of age-related changes affecting B cells^8^. In the absence of protective antibodies, SARS-CoV-2-specific T cells may afford some protection against disease progression and severity, which may be important for older individuals who fail to develop VN antibodies^9–11^. Nevertheless, like B cells, T cells also undergo age-related alterations. Thus, we investigated whether the magnitude and quality of the SARS-CoV-2-specific T cell responses were also affected by aging. To this end, we measured SARS-CoV-2-specific T cell responses by stimulating peripheral blood mononuclear cells (PBMCs) with pools of overlapping peptides spanning the SARS-CoV-2 S1 and S2 subunits of the Spike protein (homologous to the vaccine strain) using an ex vivo interferon-γ (IFN-γ) enzyme-linked immunospot (ELISpot) assay. In line with the B cell responses, the frequency of Spike-specific T cells declined with increasing age (*r* = −0.435, *P* = 0.0003) and Charlson Comorbidity Index (*r* = −0.417, *P* = 0.0005) (Fig. 1e and Extended Data Fig. 1a). No association was found between the magnitude of the Spike-specific response and time postvaccination (Extended Data Fig. 1b). Comparison of the response between age groups showed that the overall Spike-specific T cell response in individuals aged over 66 was significantly lower than in the young age group (*P* < 0.01) and, to a lesser extent (*P* < 0.05), between middle-aged and older individuals (41–65 versus ≥66 years) (Fig. 1f). Consequently, the proportion of nonresponders in the ≥66 vaccinees was higher than in the other two age groups (27.8 versus 8 versus 4.3%) (Fig. 1g). No significant difference was found between young and middle-aged individuals, indicating that impairment of the vaccine-induced immune response mainly affects older adults.

Importantly, the reduced response in individuals over 66 is most likely not due to a general defect of T cell functionality since all individuals, regardless of their age, responded equally well to stimulation with influenza virus antigens and a CD3 antibody (positive control) (Extended Data Fig. 1c,d). Moreover, the Spike-specific response in SARS-CoV-2-exposed individuals was not affected by age (Extended Data Fig. 1e). These findings suggest that age impacts the magnitude of the mRNA vaccine-elicited T cell response but does not affect preexisting memory T cells, like those specific to the influenza virus.

Most (58 out of 66, 87.9%) unexposed vaccinees mounted a T cell response to the Spike peptide pools (S1 and S2), although with a considerable heterogeneity in magnitude (17–510 spot-forming cells (SFCs) per 10^6^ PBMCs). In contrast, a low frequency of nucleocapsid and membrane protein-specific T cells was seen in 21.5% (14 out of 65) and 10.6% (7 out of 66) of the vaccinees, respectively (Fig. 1h). Only one individual with no measurable response to Spike and nucleocapsid peptide pools had a high frequency of membrane-specific T cells (114 SFCs per 10^6^ PBMCs). SARS-CoV-2 nucleocapsid and membrane antigens (not contained in the vaccine) have high sequence homology with those of seasonal human coronaviruses (HCoVs) and cross-reactive T cells to these two antigens have been reported in several studies, although their role in protection against infection is still unresolved^12–15^. Of note, their numbers inversely correlate with age^16^. As expected, most of the exposed individuals showed a response to all tested antigens: Spike 89.8% (44 out of 49); membrane, 61.2% (30 out of 49); and nucleocapsid 81.6% (40 out of 49) (Extended Data Fig. 1f).

**Fig. 2:**
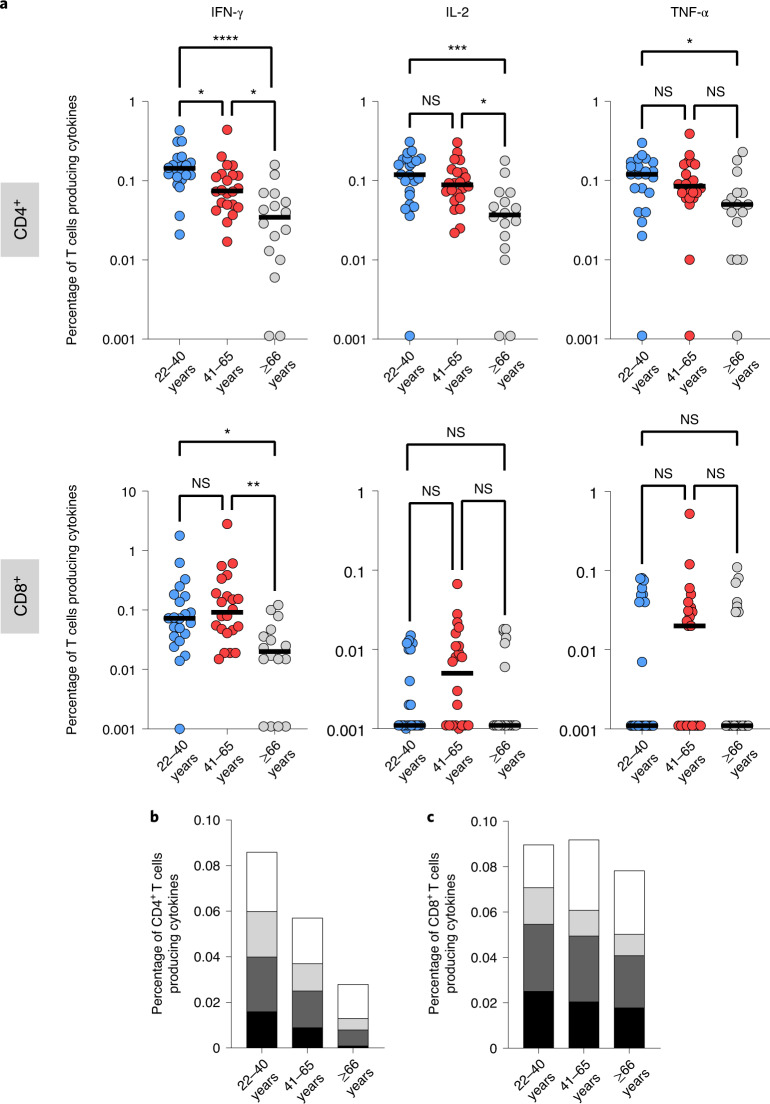
Age-dependent reduction of Spike-specific CD4^+^ and CD8^+^ T cells producing cytokines in vaccinated individuals detected by intracellular staining. **a**, The percentage of CD4^+^ (top) and CD8^+^ (bottom) T cells producing cytokines is shown for each age group (light blue, 22–40 years, *n* = 22; red, 41–65 years, *n* = 22; gray, ≥66 years, *n* = 16). **b**,**c**, Percentage of CD4^+^ T cells producing multiple cytokines (black, IFN-γ^+^/IL-2^+^/TNF-α^+^; dark gray, IFN-γ^+^/IL-2^+^; light gray, IFN-γ^+^/TNF-α^+^; white, IL-2^+^/TNF-α^+^), in ELISpot responder vaccinated (light blue, 22–40 years, *n* = 22; red, 41–65 years, *n* = 22; gray ≥66 years, *n* = 16) (**b**) and SARS-CoV-2-exposed individuals (light blue, 22–40 years, *n* = 14; red, 41–65 years, *n* = 19; gray, ≥66 years, *n* = 15) (**c**). The Spike-specific response was measured on non-naïve T cells. The gating strategy is depicted in Extended Data Fig. 2. Each dot represents a single donor and the horizontal lines indicate the medians. *P* values were determined by two-tailed Kruskal–Wallis test with Dunn’s multiple comparison correction; *P < 0.05; **P < 0.01; ***P < 0.001; ****P < 0.0001. Source data

**Extended Data Fig. 2:**
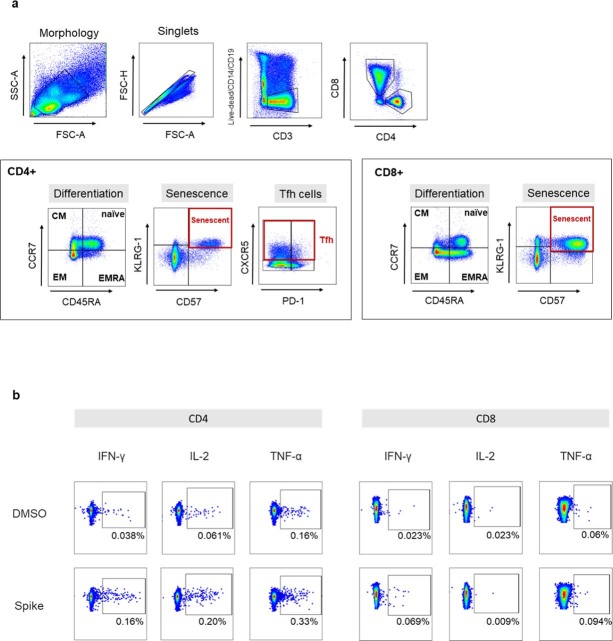
Gating strategy and examples of flow cytometry measurements. (a) Gating strategy to identify CD4 + and CD8 + T cell phenotype and cytokines production. (b) Representative flow cytometry plots identifying CD4 + (left panel) and CD8 + (right panel) producing IFN-γ, IL-2 and TNF-α in response to S peptides (bottom panel) and DMSO negative control (upper panel). Cytokines (IFN-γ, IL-2 and TNF-α) were analyzed on non-naïve (memory) T cells.

**Extended Data Fig. 3:**
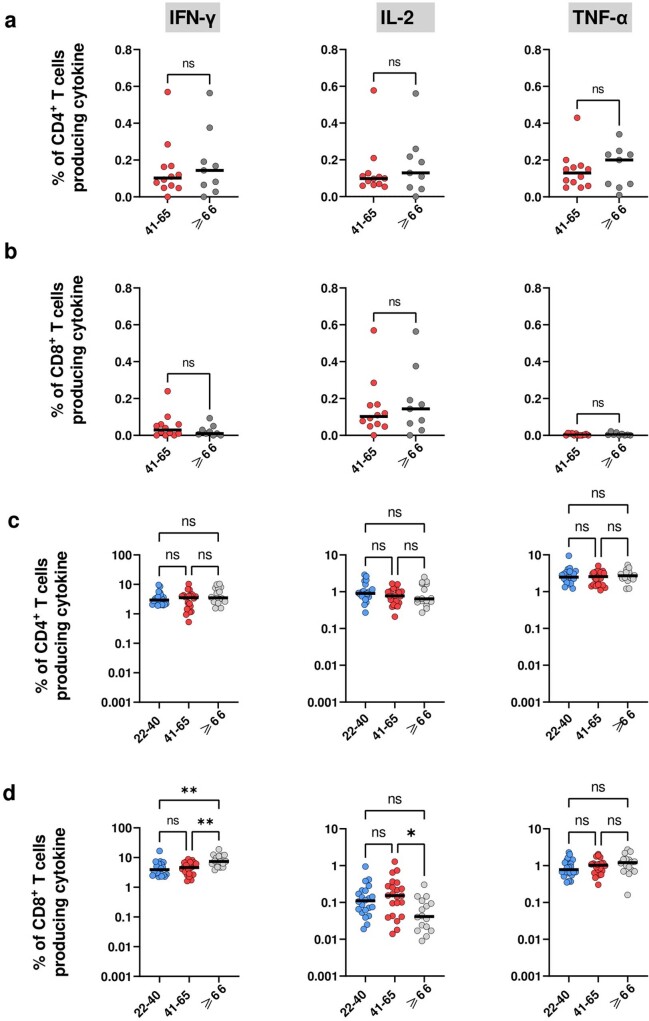
Intracellular cytokines secretion by SARS-CoV-2 exposed or vaccinated subjects upon spike-specific or CD3-specific stimulation, respectively. Intracellular IFN-γ, IL-2 and TNF-α cytokines secretion by (a) CD4 + and (b) CD8 + T cells in response to S stimulation in 41–65 (red; n = 12) and ≥66 (grey; n = 9) years SARS-CoV-2 exposed subjects, 50–100 days post SARS-CoV-2 infection. Cytokines production by (c) CD4 + (upper) and (d) CD8 + (lower) T cells from vaccinated subjects and stimulated via CD3 engagement (n = 63). Spike- and CD3-specific responses were analysed on non-naïve T cells. Each dot represents a single study subject and horizontal lines indicate medians. P values were determined by two-tailed Kruskal-Wallis test with Dunn’s multiple comparison correction (3 age groups comparison) or Mann-Whitney for comparison of two groups; * P < 0.05; ** P < 0.01; ns: not significant; yr: years. Source data

It has been shown that the mRNA-based COVID-19 vaccine induces Spike-specific CD4^+^ and CD8^+^ T cells^17^. Because the ELISpot assay does not allow identification of T cell subsets, we utilized intracellular cytokine staining (ICS) and flow cytometry to further characterize the responding cells. PBMCs were stimulated with the Spike peptide pool and CD3^+^CD4^+^ and CD3^+^CD8^+^ non-naïve T cells were analyzed for the production of IFN-γ, interleukin-2 (IL-2) or tumor necrosis factor-α (TNF-α). The gating strategy is depicted and representative examples of ICS of CD4^+^ and CD8^+^ cells after stimulation with Spike peptides are shown in Extended Data Fig. 2a,b. IFN-γ-producing CD4^+^ and CD8^+^ T cells were detected on mRNA vaccination and, in agreement with the results obtained with the IFN-γ ELISpot assay, although with a higher magnitude when measured by ICS, the Spike-specific response was significantly lower in older adults, compared to young adults (CD4^+^ *P* < 0.0001; CD8^+^ *P* < 0.05) and middle-aged individuals (CD4^+^ *P* < 0.05; CD8^+^ *P* < 0.01) (Fig. 2a). Younger adults displayed a statistically significant higher frequency of Spike-specific CD4^+^IFN-γ^+^ cells than middle-aged adults (*P* < 0.05), which was not observed for CD8^+^ T cells. Similarly, age-dependent differences were found for IL-2^+^ (22–40 versus ≥66, *P* < 0.001; 41–65 versus ≥66, *P* < 0.05; 22–40 versus 41–65, not significant) and TNF-α^+^ CD4^+^ T cells (22–40 versus ≥66, *P* < 0.05; 41–65 versus ≥66, and 22–40 versus 41–65 not significant) (Fig. 2a). No differences were found between age groups in relation to CD8^+^ T cells producing IL-2 and TNF-α since IFN-γ dominates the Spike-specific response in vaccinees (Fig. 2a). Noteworthy, comparison of individuals aged 41–65 and ≥66 years exposed to SARS-CoV-2 showed no age-dependent differences in Spike-specific non-naïve CD4^+^ and CD8^+^ T cells producing IFN-γ, IL-2 or TNF-α (Extended Data Fig. 3a,b). Due to the small numbers, we could not include young adults in this comparison. PBMCs from vaccinated older individuals stimulated via CD3 engagement showed similar CD4^+^ T cell responses to those detected in 22–40- and 41–65-year-old individuals (Extended Data Fig. 3c). The frequency of IFN-γ-producing CD8^+^ T cells in response to CD3 increased with age (22–40 versus ≥66, *P* < 0.01; 41–65 versus ≥66, *P* < 0.01), but not those producing IL-2 (41–65 versus ≥66, *P* < 0.05) (Extended Data Fig. 3d).

**Extended Data Fig. 4:**
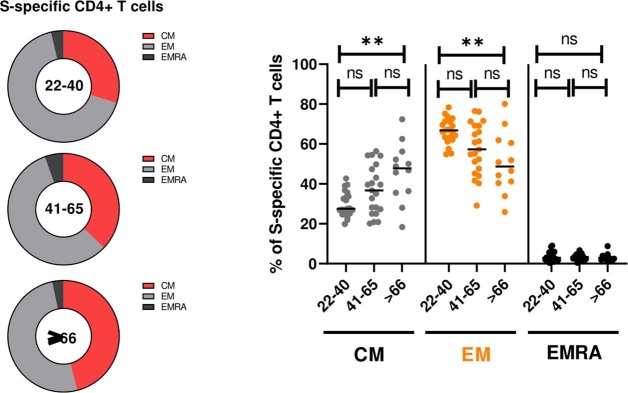
Distribution of the vaccine-induced S-specific CD4 + T cells among differentiation phenotypes. Distribution of the vaccine-induced S-specific CD4 + T cells among differentiation phenotypes. Distribution of the vaccine-induced S-specific CD4 + T cells among central memory (CM), effector memory (EM) and effector memory CD45RA + (EMRA) phenotype (22–40 yr, n = 21; 41–65 yr, n = 21; ≥ 66 yr, n = 12). S-specific response is determined based on cytokines production on non-naïve CD4 + T cells. Each dot represents a single study subject and horizontal lines indicate medians. P values were determined by two-tailed Kruskal-Wallis test with Dunn’s multiple comparison correction (3 age groups comparison); ** P < 0.01; ns: not significant. Source data

In line with these findings, we also observed an age-dependent reduction of CD4^+^ T cells producing two (IL-2^+^IFN-γ^+^, TNF-α^+^IFN-γ^+^ and IL-2^+^TNF-α^+^) and three cytokines (IFN-γ^+^, IL-2^+^, TNF-α^+^) in response to Spike peptide stimulation, with older individuals showing a remarkable decrease of those polyfunctional T cells compared to young adults (67.4%) and the middle-aged group (50.8%) (Fig. 2b). Of interest, a reduction of CD4^+^ polyfunctional T cells could already be observed in middle-aged individuals compared to young adults (33.7%, 41–65 versus 22–40 years) (Fig. 2b). In contrast, SARS-CoV-2 infection induced polyfunctional CD4^+^ T cells in all age groups, although to a slightly lower extent in older adults (Fig. 2c). Furthermore, the frequency of polyfunctional CD4^+^ T cells was similar in vaccinated (0.086%) and SARS-CoV-2-infected (0.089%) younger adults indicating that infected and vaccinated individuals were otherwise comparable (Fig. 2b,c). We investigated the differentiation stage of the non-naïve Spike-specific CD4^+^ T cells in vaccinees and found an age-dependent redistribution of these cells within the central memory T (T_CM_) and effector memory T (T_EM_) cells (Extended Data Fig. 4). Younger and middle-aged individuals showed a higher percentage of T_EM_ over T_CM_ cells, whereas in the group of older adults the frequency of Spike-specific T_CM_ and T_EM_ cells was comparable. No differences were found for the effector memory CD45RA^+^ T (T_EMRA_) cells.

In summary, these data demonstrated that in older adults, COVID-19 mRNA vaccination elicited a lower frequency of Spike-specific CD4^+^ and CD8^+^ T cells producing cytokines involved in T cell differentiation and proliferation. Furthermore, polyfunctional T cells are involved in protective immunity to virus infections and their low numbers in older adults may contribute to suboptimal protection provided by vaccination in this age group^18^.

**Fig. 3:**
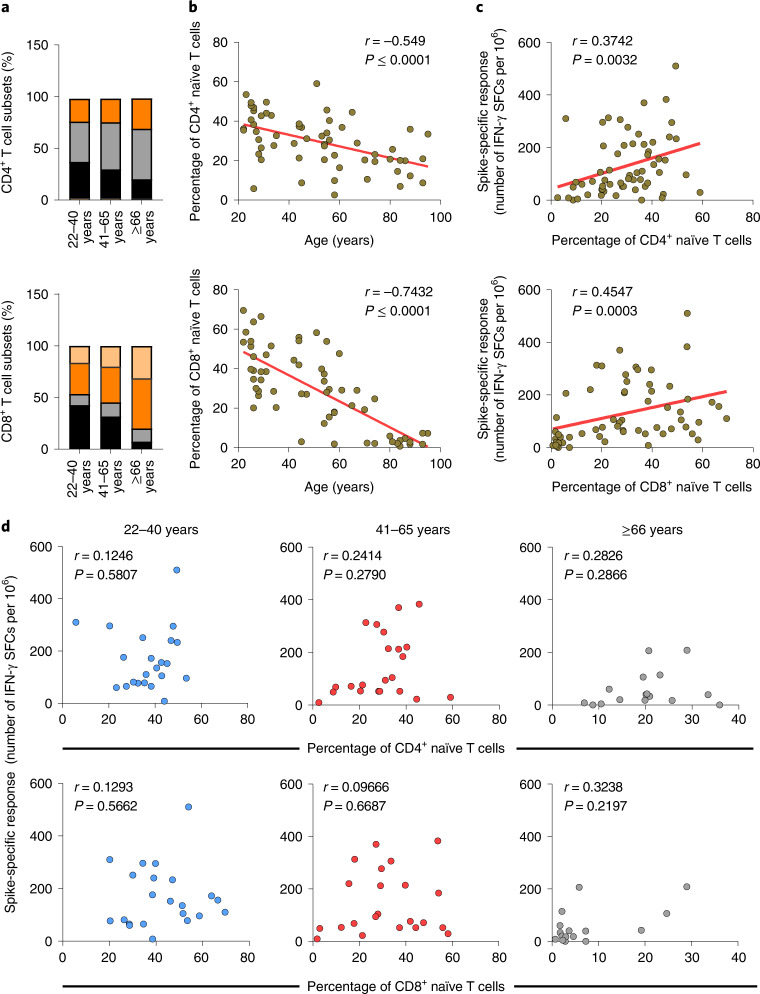
The lower vaccine-induced, Spike-specific T cell response in older adults correlates with a reduced frequency of CD4^+^ and CD8^+^ naïve T cells. **a**, CD4^+^ (upper) and CD8^+^ (lower) T cell differentiation subsets (naïve, black; T_CM_, gray; T_EM_, orange; T_EMRA_, light orange) in vaccinated individuals (*n* = 60). **b**, Correlation between age and percentage of CD4^+^ (upper, *r* = −0.549; *P* < 0.0001) or CD8^+^ (lower, *r* = −0.743; *P* < 0.0001) naïve T cells. **c**, Spike-specific IFN-γ response association with percentage of CD4^+^ (*r* = 0.374; *P* = 0.0032) or CD8^+^ (*r* = 0.454, *P* = 0.003) naïve T cells. **d**, Spike-specific response in correlation with the percentage of naïve CD4^+^ (upper) and CD8^+^ (lower) T cells for each age group. Each dot represents a single donor. **b**–**d**, A two-tailed Spearman’s test was used to the test the significance (*r* and *P* values). **b**–**c**, The red line represents the linear regression. The gating strategy is depicted in Extended Data Fig. 2. Source data

**Extended Data Fig. 5:**
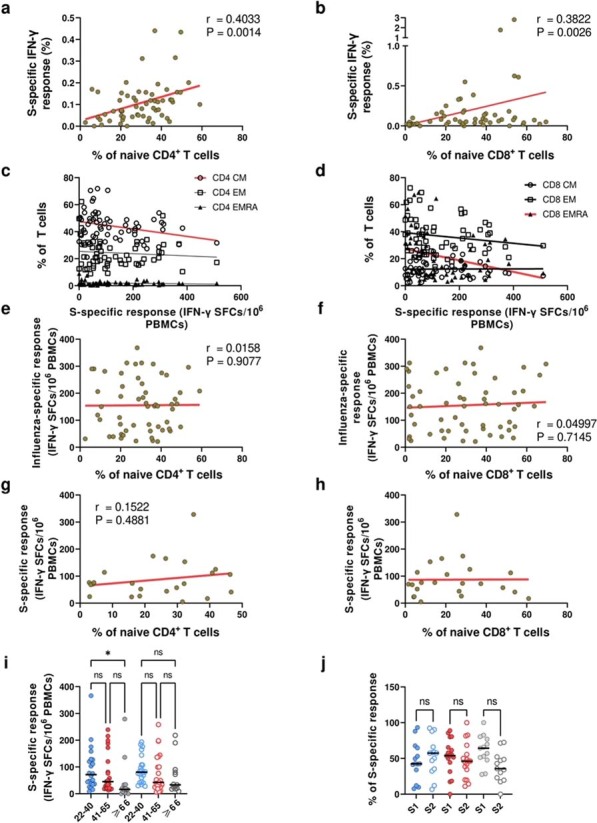
Correlations between spike- or influenza-specific response and frequency of differentiation subsets, and specificity of the spike response. (a) Correlation between S-specific IFN-γ response of non-naïve CD4 + (r = 0.403, P = 0.001; n = 60) or (b) CD8 + T cell (r = 0.382, P = 0.0026; n = 60) with percentage of naïve T cells in vaccines, measured by FACS. Correlation of percentage of (c) CD4 + (n = 60) and (d) CD8 + (n = 60) central memory (CM), effector memory (EM), and effector memory RA + (EMRA) with number of S-specific IFN-γ SFCs. Correlation of IFN-γ production (SFCs) in response to influenza virus (n = 56) in (e) CD4 + and (f) CD8 + naïve T cells. Correlation of S-specific IFN-γ SFCs with (g) CD4 + and (h) CD8 + naïve T cells in exposed subjects 50–100 days post positive PCR. (i) Frequency of S1 (filled circle) or S2 (open circle) specific IFN-γ SFCs in vaccinated subjects responding to S protein (light blue 22–40 ys n = 22; red 41–65 ys n = 23; grey ≥66 yr, n = 13). (j) Proportion of T cells directed to the S1 (filled circle) or S2 (open circle) region of the spike protein in SARS-CoV-2 exposed subjects (light blue 22–4 ys n = 13; red 41–65 ys n = 17; grey ≥66 yr, n = 14). Each dot represents a single study subject and horizontal lines indicate medians. The red line represents linear regression and two-tailed Spearman’s test was used to the test the significance (r and P values). P values were determined by two-tailed Kruskal-Wallis test with Dunn’s multiple comparison correction; * P < 0.05; ns: not significant; yr: years. Source data

Because T cell populations are reshaped during aging we sought to investigate whether the differentiation status of circulating T cells correlated with the magnitude of the immune response induced by mRNA vaccination^6,19^. To this end, we defined four differentiation subsets of CD4^+^ and CD8^+^ T cells based on the surface expression of the CD45RA and CCR7 molecules (naïve, T_CM_, T_EM_ and T_EMRA_) and correlated the proportion of these cells with the frequency of Spike-specific T cells, measured by IFN-γ ELISpot. Overall, we found a decrease of naïve and an accumulation of terminally differentiated T cells, as described previously (Fig. 3a)^6^. This age-dependent reduction of naïve CD4^+^ and CD8^+^ T cells, which was more profound in the CD8^+^ compartment (CD4^+^ *r* = 0.549, *P* < 0.0001 upper; CD8^+^ *r* = 0.743, *P* < 0.0001 lower) (Fig. 3b), correlated with reduced numbers of Spike-specific IFN-γ SFCs (CD4^+^ *r* = 0.374, *P* = 0.0032 and CD8^+^ *r* = 0.454, *P* = 0.0002) (Fig. 3c). Comparable results were obtained when the IFN-γ response was measured on non-naïve T cells by ICS (Extended Data Fig. 5a,b). Comparison of the results obtained in the respective age groups showed an age-dependent association between CD4^+^ and CD8^+^ naïve T cells and the Spike-specific T cell response (Fig. 3d). Especially the loss of naïve CD8^+^ T cells in individuals aged ≥66 was inversely correlated with the vaccine-induced T cell response. Of note, no correlation was found with the CD8^+^ T_CM_ and T_EM_ subsets and CD4^+^ T_EM_ and T_EMRA_, while a higher frequency CD4^+^ T_CM_ and CD8^+^ T_EMRA_ in older individuals correlated inversely with the vaccine-induced T cell response (Extended Data Fig. 5c,d). Remarkably, the proportion of naïve CD4^+^ and CD8^+^ T cells did not correlate with the frequency of T cells directed against the influenza virus, for which immunological memory exists and can be recalled in vitro^20,21^ (Extended Data Fig. 5e,f). There was also no correlation with the Spike-specific responses measured in the exposed individuals (Extended Data Fig. 5g,h). Comparison of the three age groups confirmed that there is an age-dependent inverse correlation between naïve CD4^+^ and especially CD8^+^ T cells on the one hand, and the magnitude of the T cell response measured by IFN-γ ELISpot assay on the other. Thus, the lower magnitude of Spike-specific response on COVID-19 mRNA vaccination in older adults correlated with decreased numbers of naïve T cells. This suggests that this subset of T cells may play a role in the COVID-19 vaccination outcome because induction of a primary response to new antigens mainly relies on the activation of naïve T cells^22,23^. Of note, a low frequency of naïve T cells has been associated with more severe COVID-19 disease and impaired priming of naïve CD8^+^ T cells in older adults^9,24^. Moreover, restricted T cell receptor diversity and altered signaling in naïve T cells may generate a less effective pool of memory cells, which could lead to a suboptimal response, exposing older adults at higher risks of infection and disease severity^19,25,26^.

**Fig. 4:**
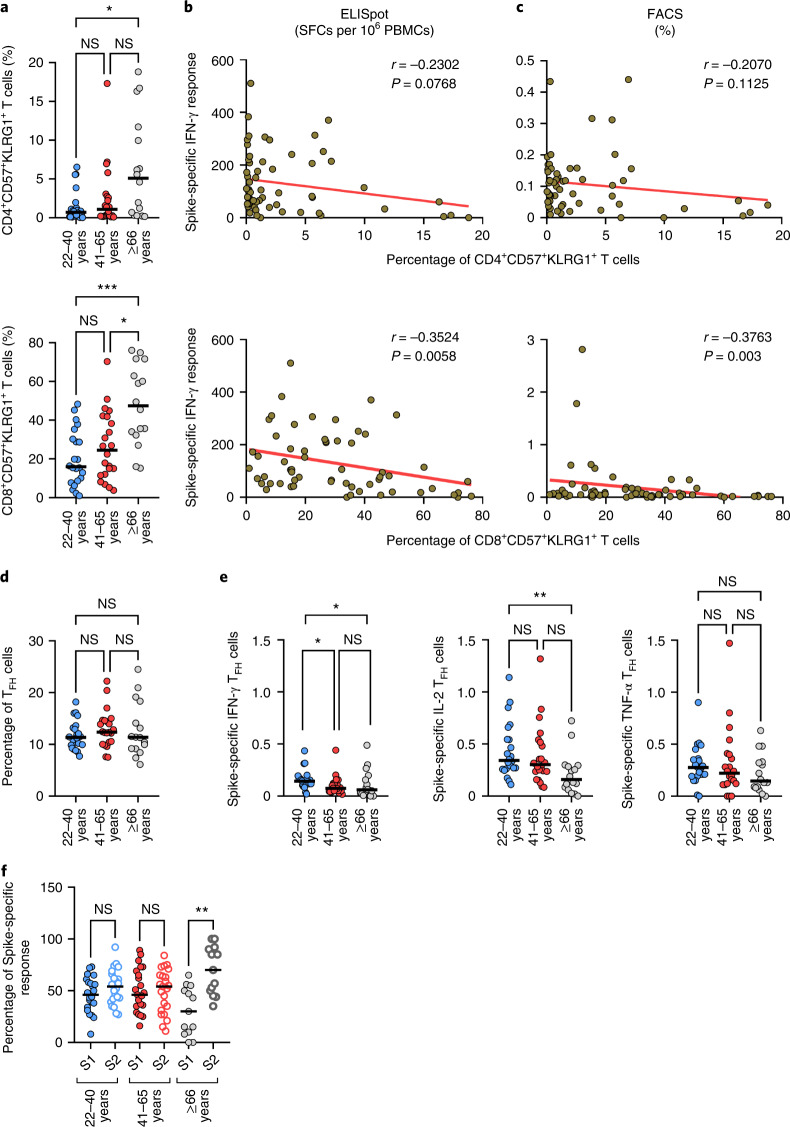
Lower vaccine-induced, Spike-specific T cells in older adults correlate with increased frequency of immunosenescent T cells, reduced frequency of T_H_1 and T_FH_ cells and skewed response to the S2-region of the Spike protein. **a**–**c**, Frequency of CD57^+^KLRG1^+^ senescent CD4^+^ (upper) and CD8^+^ (lower) T cells for each age group (light blue, 22–40 years, *n* = 22; red, 41–65 years, *n* = 22; gray, ≥66 years, *n* = 16) (**a**) and their correlation with Spike-specific IFN-γ response measured by ELISpot (CD4^+^, not significant; CD8^+^, *r* = −0.352, *P* = 0.0058) (**b**) or flow cytometry (CD4^+^, not significant; CD8^+^, *r* = −0.376, *P* = 0.003) (**c**). **d**, Frequency of circulating T_FH_ cells in the different age groups (light blue, 22–40 years, *n* = 22; red, 41–65 years, *n* = 22; gray, ≥66 years, *n* = 16), identified based on the expression of CD4^+^ and the homing receptor CXCR5. **e**, Frequency of Spike-specific T_FH_ cells producing IFN-γ, IL-2 or TNF-α in vaccinated individuals (light blue, 22–40 years, *n* = 22; red, 41–65 years, *n* = 22; gray, ≥66 years. *n* = 16). **f**, Proportion of T cells directed to the S1 (filled circle) or S2 (open circle) region of the Spike protein in IFN-γ responder individuals (light blue, 22–40 years, *n* = 22; red, 41–65 years, *n* = 23; gray, ≥66 years, *n* = 13). Spike-specific response of circulating T_FH_ cells was analyzed on non-naïve CD4^+^ T cells. The gating strategy is depicted in Extended Data Fig. 2. Each dot represents a single study individual and the horizontal lines indicate the medians. The red line represents the linear regression and a two-tailed Spearman test was used to the test the significance (*r* and *P* values). *P* values were determined by a two-tailed Kruskal–Wallis test with Dunn’s multiple comparison correction; *P < 0.05; **P < 0.01; ***P < 0.001. Source data

**Extended Data Fig. 6:**
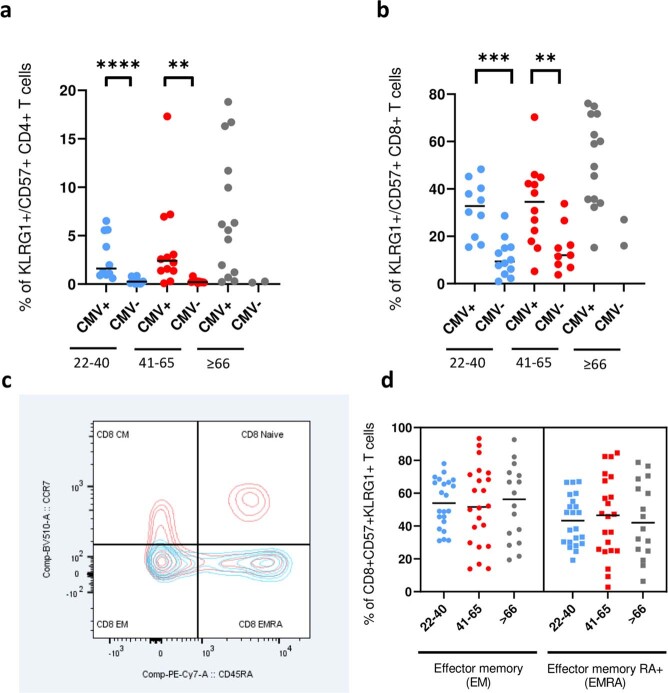
Distribution of T cells expressing senescent markers. Distribution of KLRG1 + CD57 + senescent (a) CD4 + and (b) CD8 + T cells among CMV positive (CMV + ) and negative (CMV-) in 22–40 (CMV + n = 10; CMV- n = 12) and 41–65 (CMV + n = 12; CMV- n = 9) and ≥66 (CMV + n = 14; CMV- n = 2) years old subjects. (c) Example of FACS contour plot depicting the distribution of the CD57 + KLRG1 + senescent CD8 + T cells (light blue) on total CD8 + T cell differentiation subsets (red). (d) Distribution of the CD8 + senescent cells within the EM (circle) and EMRA (square) differentiation subsets in all age groups (light blue: 22–40 yr, n = 22; red: 41–65 yr, n = 22; grey ≥ 66 yr, n = 16). Each dot represents a single study subject and horizontal lines indicate medians. P values were determined by two-tailed Mann-Whitney for comparison of two groups. Source data

To unveil other potential age-dependent factors that may affect the response to vaccination in older people, we also characterized the magnitude and effector functions of terminally differentiated T cells coexpressing the CD57 and KLRG1 molecules, which have been associated with aging^23,27^. Despite a general age-dependent increase of CD4^+^ (Fig. 4a, upper panel) and CD8^+^ (Fig. 4a, lower panel), T cells coexpressing these senescence markers, only the CD8^+^ cells were inversely correlated with the magnitude of the Spike-specific IFN-γ T cell response measured by ELISpot (*r* = −0.352, *P* = 0.0058; Fig. 4b) and fluorescence-activated cell sorting analyses (*r* = −0376, *P* = 0.003; Fig. 4c), although such correlation does not necessarily imply causality. Of note, some younger individuals showed a high proportion of CD4^+^ and CD8^+^ T cells coexpressing the senescence markers; interestingly, that correlated with the presence of serum antibodies to CMV (Extended Data Fig. 6a,b). This finding supports the notion that chronic infections, like those caused by cytomegalovirus (CMV), may be responsible for the accumulation of CD57^+^ KLRG1^+^ T cells, regardless of age. We found that the CD8^+^ T cells coexpressing such markers were of the T_EM_ and T_EMRA_ phenotype and were similarly distributed between age groups (Extended Data Fig. 6c,d). No correlation was found with the percentage of CD4^+^ CD57^+^ KLRG1^+^ T cells.

Next, since we observed reduced B cell responses (antibodies and memory cells) in older vaccinees, we addressed the question whether alterations of circulating follicular helper T (T_FH_) cells related to reduced antibody responses^28^. Interestingly, the circulating T_FH_ cells (CD3^+^CD4^+^CXCR5^+^) were numerically similar between the age groups (Fig. 4d). However, when we analyzed the Spike-specific non-naïve circulating T_FH_ cells, IFN-γ and IL-2 production was lower in the ≥66 group (IFN-γ, *P* < 0.05; IL-2, *P* < 0.01) compared to that of young adults (Fig. 4e). Of note, measurement of IFN-γ, IL-2 and TNF-α might have underestimated the frequency of the Spike-specific circulating T_FH_ cells as other markers (for example, ICOS and IL-21) may be expressed by a larger proportion of circulating T_FH_.

No differences were found for the TNF-α-producing circulating T_FH_ cells (Fig. 4e). Of note, a high frequency of T_H_1-like circulating T_FH_ correlated with strong antibody responses to influenza and other viruses^29–31^.

We then investigated if, apart from the magnitude of the Spike-specific T cell response, age influenced the specificity of the T cell response. To this end, we tested the response to peptide pools spanning the S1 and S2 regions of the Spike protein, respectively. A similar frequency of S2-specific T cells was observed in all age groups (Extended Data Fig. 5i). However, the frequency of S1-specific T cells was significantly lower in older adults compared to young adults (*P* < 0.05). Moreover, when we calculated the relative contribution of the S1- and S2-specific response in the individuals who displayed a Spike-specific response, we noticed that older adults had a preferred response to the S2 region (*P* < 0.01) (Fig. 4f). This is in contrast with middle-aged and young adults, who had similar responses to S1 and S2 (Fig. 4f). In the SARS-CoV-2-exposed individuals, no statistically significant difference was observed (Extended Data Fig. 5j). S2 is more conserved than S1 across coronaviruses; therefore, it may represent a target of preexisting cross-reactive memory B and T cells originally induced by previous infections with ‘common cold’ HCoVs^32^.

Together with the reduced number of naïve T cells leading to suboptimal responses to a new antigen like the S1 region, this may explain the preferential recognition of the S2 region in older vaccinees^33^.

Collectively, our data provide insights into the age-dependent immunological changes that may account for the reduced B and T cells responses observed in older adults on BNT162b2 vaccination; however, interestingly, this is not the case with the SARS-CoV-2 infection. The different antigenic load, anatomical site of antigen encounter (intramuscular versus mucosal), strength and length of T cell receptor engagement (replicating virus versus mRNA-encoded antigen), multiple viral antigens (SARS-CoV-2) versus mRNA-encoded Spike protein, may all account for the differential responses observed in this study. New vaccine approaches or use of substances able to overcome age-related defects would help to provide optimal protection to this vulnerable age group.

Our data suggest a general age-dependent decrease of the adaptive immune responses on BNT162b2 vaccination. However, our study has some limitations. The study was designed as a large cross-sectional epidemiological study and we have used samples from a subset of study individuals to investigate the SARS-CoV-2-specific immune response on vaccination and infection. Therefore, our cohort is heterogeneous and no correction for age and sex was performed since this would have required a larger sample size. Furthermore, the limited number of PBMCs available precluded the possibility to analyze the additional effector functions and biomarkers of the immune cells possibly associated with aging and immunosenescence. The correlation found between aging and redistribution of the T cell differentiation subsets and suboptimal T and B cell immune responses on vaccination do not imply a direct causality since some of the investigated variables (for example, CD8^+^ T cells with senescent phenotype) correlate with age.

## Methods

### Study participants and ethics statement

This study complies with all relevant ethical regulations. An ethical approval was given by the ‘Aerztekammer Niedersachsen’ (Bo/31/2010 amended in November 2021) and written informed consent was obtained from every individual or legal representant.

### Characteristics of the study cohort and sample collection

A total of 115 participants were enrolled in this study: 66 SARS-CoV-2 with no history of infection received two doses of the BNT162b2 vaccine (Comirnaty; BioNTech/Pfizer); 49 laboratory-confirmed (PCR and antibodies) convalescent individuals with COVID-19 with a mild or asymptomatic SARS-CoV-2 infection were part of a larger prospective epidemiological study, whose data were published previously^7^. The Charlson Comorbidity Index was calculated based on the information collected with a questionnaire at the time of enrollment^34^. Full cohort and demographic information are provided in Supplementary Tables 1 and 2. Blood samples were taken once between 21 and 263 d (median 79 d) post-positive PCR and 40–81 d (median 44 days) after the first vaccination (Supplementary Table 1). No statistical methods were used to predetermine sample sizes; nevertheless, our sample sizes are similar to those reported in previous publications^35–38^. Data collection and analysis were not performed blind to the conditions of the experiments. No compensation was provided to participants. Serum was obtained by centrifuging (room temperature) the whole blood at 4,000 r.p.m. for 15 min and stored at −20 °C. Blood samples for cellular analyses were collected into sodium heparin and EDTA tubes (Sarstedt) and PBMCs were isolated by density gradient centrifugation using Lymphoprep (STEMCELL Technologies) according to the manufacturer’s protocol. Cells were frozen in freezing medium containing 90% FCS (Thermo Fisher Scientific) and 10% dimethyl sulfoxide (DMSO) (Sigma-Aldrich) and stored at −150 °C until further use. Roswell Park Memorial Institute (RPMI) 1640 (Thermo Fisher Scientific) supplemented with GlutaMAX, penicillin/streptomycin, nonessential amino acids, vitamins, sodium pyruvate (Thermo Fisher Scientific) and 10% heat-inactivated FCS (hereafter R10F) containing 50 µg ml^−1^ DNase (Sigma-Aldrich) was used for thawing the PBMCs. R10F was used for cell culture and dilution of stimulating reagents. For all samples, cell viability was >85%.

### Virus neutralization assay

Neutralizing antibodies were evaluated in all the individuals tested as described previously^7^. In brief, inactivated serum samples (56 °C, 30 min) were first diluted 1:10 followed by 1:2 serial dilutions. Then, 50 µl of the diluted sera was mixed with 50 µl of a SARS-CoV-2 Wuhan-Hu-1 dilution with a concentration of 4,000 TCID_50_ ml^−1^ and incubated for 1 h at 37 °C. Serum-virus mix was added to a monolayer of Vero cells and further incubated at 37 °C, 5% CO_2_. After 8 h of incubation, cells were fixed using 4% paraformaldehyde (PFA) and incubated for 30 min at room temperature. Then, PFA was removed and cells were incubated for 15 min with 80% methanol. Furthermore, plates were blocked using 1% BSA in PBS-0.05% Tween 20 for 30 min at 37 °C. SARS-CoV-2 was detected using a 1:1,000 dilution of rabbit polyclonal anti-SARS-CoV-2 nucleocapsid (SinoBiological). After 1 h incubation at 37 °C, cells were washed with PBS-0.05% Tween 20 and incubated with a 1:1,000 dilution of anti-rabbit-IgG-Alexa Flour 488 (Invitrogen). Finally, cells were washed twice with PBS-0.05% Tween 20. Fluorescent cells were counted using the CTL S6 Ultimate-V Analyzer and data were analyzed using the CTL ImmunoSpot version 7.0.20.0 software. Neutralizing antibody titers are expressed as the dilution that gave a 50% reduction of stained cells.

### Detection of memory B cells by ELISpot

A total of 1–2 × 10^6^ PBMCs were seeded in duplicate in a 48-well plate in the presence of R848 (1 µg ml^−1^; Mabtech) and IL-2 (10 ng ml^−1^; Mabtech) for 72 h at 37 °C and 5% CO_2_. MultiScreenHTS-HA plates (Merck Millipore) were coated with 100 µl per well of recombinant Spike protein (rS) (SARS-CoV-2 Spike Trimer HEK; Miltenyi Biotec) at 5 µg ml^−1^ in sterile PBS. PBS and a mixture of anti-kappa/anti-lambda antibodies (6 µg ml^−1^ each; SouthernBiotech) was used as negative or positive controls, respectively. After overnight incubation at 4 °C, the plates were washed 3 times with 200 µl per well of sterile PBS (Gibco) and blocked with 200 µl per well of R10F for at least 30 min at room temperature. Stimulated cells were washed in R10F and seeded at 2.5 × 10^5^ in the PBS and rS wells, while 2.5 × 10^4^ of the cells was used for the kappa/lambda (k/l) positive control, all in triplicate or duplicate in case of limited number of cells. After 4 h at 37 °C and 5% CO_2_, plates were washed 5 times with PBS-Tween 0.05% and IgG-horseradish peroxidase (HRP) and IgA-AP detection antibodies (SouthernBiotech) at 1:900 added for 1 h at room temperature. Spots were developed after washing and stepwise addition of substrate solutions for ALP- (BCIP/NBT-plus; Mabtech) and HRP-conjugated antibodies (AEC; Mabtech), as described earlier^39^. Plates were then scanned and antibody-secreting cells counted using the ImmunoSpot S6 Ultimate Reader and ImmunoSpot Software v.7.0.20.0 (ImmunoSpot; CTL). Data were calculated using the mean of replicate wells and expressed as SFCs per million of in vitro expanded PBMCs after subtracting the PBS control values. The percentage of Spike-specific MBCs was calculated by dividing the number of Spike-specific IgG MBCs over total number of IgG MBCs.

### Synthetic peptides

Two pools containing 158 (S1) and 155 (S2) 15-mers with 11-amino acid overlap peptides (>90% purity) and covering the amino acid residues 1–1,273 of the SARS-CoV-2 Spike protein (protein ID: P0DTC2; catalog no. RP30027; GenScript); pepmixes of 15-mer peptides overlapping by 11 amino acid residues covering the nucleocapsid protein (Swiss-Prot ID: P0DTC9; JPT) and membrane (GenBank ID: QHO60597.1; catalog no. NR-52403, BEI Resources; GenPept ID: QHO60597) peptide pools of SARS-CoV-2 contained 17-, 13- or 12-mer peptides with 10-amino acid overlaps and spanned the whole protein sequences, were used for the study. All peptides were reconstituted in high-grade DMSO (Sigma-Aldrich) and further diluted according to the manufacturer’s recommendations (stock solution 50 µg ml^−1^). The final concentration of DMSO in culture was below 0.5%.

### IFN-γ ELISpot assay

Precoated 96-well plates were purchased from Mabtech and the assay was carried out according to the manufacturer’s instructions. Briefly, plates were washed with sterile PBS (Thermo Fisher Scientific) and blocked with R10F for at least 1 h. Then, 2.5 × 10^5^ (SARS-CoV-2 and influenza) or 2.5 × 10^4^ (CD3, positive control) PBMCs per well were stimulated in triplicates with overlapping peptide pools at a concentration of 0.7 µg ml^−1^ for SARS-CoV-2 S1, S2 and nucleocapsid or 0.4 µg ml^−1^ for membrane per individual peptide. Influenza vaccine (season 2020–2021; 1 µg ml^−1^ per hemagglutinin; Vaxigrip Tetra Sanofi) and anti-CD3 antibody (1:1,000; Mabtech) were used as controls. In some cases (limited number of PBMCs), the ELISpot was run in duplicate. Negative control comprised equimolar amounts of DMSO. Plates were incubated for 20 h, plates developed and spots counted using the ImmunoSpot S6 Ultimate Reader equipped with the ImmunoSpot Software. The mean spot counts for the DMSO negative control were subtracted from the mean of the SARS-CoV-2 and influenza or CD3-stimulated cells. Data are displayed as SFCs per million of PBMCs. The cutoff response in the test cohort was determined using the mean from all individuals in the DMSO negative control + 2 s.d. A response >15 SFCs per 10^6^ PBMCs was considered positive.

### ICS

A total of 2 × 10^6^ PBMCs were stimulated for 20 h at 37 °C, 5% CO_2_ in a 96-well round-bottom plate using a SARS-CoV-2 Spike peptide pool (S1 + S2; 0.5 µg per ml peptide) As positive control, cells were stimulated with purified anti-CD3 (0.1 µg ml^−1^; BD Biosciences), while an equimolar amount of DMSO was used as the negative control. Costimulatory anti-CD28 and anti-CD49d purified antibodies (1 µg ml^−1^ each; BD Biosciences) were added to all wells and Brefeldin A (7 µg ml^−1^; Sigma-Aldrich) was included for the last 4 h of incubation. Cells were first washed with PBS, stained for 20 min at room temperature with LIVE/DEAD Fixable Near-IR stain kit (Molecular Probes) followed by 2 washes with PBS. After Fc receptor blocking for 20 min (Fc Block; BD Biosciences) antibodies for surface staining were added and incubated for 20 min (Supplementary Table 3). Next, cells were washed again, fixed/permeabilized using Cytofix/Cytoperm solution (BD Biosciences) according to the manufacturer’s instructions. After two washes with PBS and Perm/Wash buffer (BD Biosciences), cells were blocked for 20 min with Fc Block; then, antibodies were diluted in Perm/Wash buffer incubated for an additional 20 min (Supplementary Table 3). Cells were washed twice and resuspended in PBS. All incubations were performed at room temperature in the dark. An average of 3 × 10^5^ events per sample were acquired on a BD LSRFortessa X-20 (BD Biosciences). Cytometer setup and tracking beads (BD Biosciences) were used to define the baseline performance of the cytometer. Compensation matrix was performed using OneComp eBeads (Invitrogen). All data were analyzed using FlowJo v.10.8.1 (FlowJo LLC). Spike-, influenza- and CD3-specific responses (phenotype and cytokines) were calculated by gating on non-naïve CD4^+^ and CD8^+^ T cells; displayed values were subtracted from the DMSO control. The nonantigen-specific T cell phenotype (differentiation markers, senescent and T_FH_ cell) was analyzed on DMSO-stimulated PBMCs. Data on double- or triple-producing IFN-γ/IL-2/TNF-α cells were obtained using Boolean gating on single cells positive for each cytokine. The gating strategy is shown in Extended Data Fig. 2a.

### CMV serology

Sera samples stored at −20 °C were thawed at room temperature. Dilution and further steps were performed according to the manufacturer’s instructions (CMV IgG ELISA Kit; Tecan). Briefly, serum samples were diluted 1:100 and added to a 96-well plate precoated with CMV antigens, followed by 1-h incubation at 37 °C. After 3 washes, peroxidase-labeled anti-IgG was added and incubated for 30 min at room temperature in the dark. On washing, 3,3′,5,5′-tetramethylbenzidine substrate solution was added to the wells and incubated for 15 min; the color reaction was stopped by adding 0.2 mol l^−1^ sulfuric acid. Absorbance was measured at 450/620 nm using a Tecan SPARK Microplate Reader. Data were validated by using negative, positive and cutoff controls, as well as blanks.

### Statistics and reproducibility

Descriptive and significance statistics and displaying the data were done using Prism 9 (GraphPad Software) and Excel (Microsoft Excel 2016). Statistical analyses were performed using nonparametric tests. Pairwise correlations were determined using two-tailed Spearman tests. Kruskal–Wallis tests, with subsequent Dunn’s multiple comparison tests, were used to compare the age groups. *P < 0.05, **P < 0.01, ***P < 0.001 and ****P < 0.0001.

### Reporting summary

Further information on research design is available in the Nature Research Reporting Summary linked to this article.

## Supplementary information

Supplementary Information Supplementary Tables 1–3

Reporting Summary

## Data Availability

Raw.fcs files have been uploaded to the ImmPort repository (https://www.immport.org/shared/home; access no. SDY1961). Source data for Figs. 1–4 and Extended Data Figs. 1 and 3–6 are provided with this paper.

## Source data

Source Data Fig. 1
Source Data Fig. 2
Source Data Fig. 3
Source Data Fig. 4
Source Data Extended Data Fig. 1
Source Data Extended Data Fig. 3
Source Data Extended Data Fig. 4
Source Data Extended Data Fig. 5
Source Data Extended Data Fig. 6
