# Supplementary material for: Signs of immunosenescence correlate with poor outcome of mRNA COVID-19 vaccination in older adults
Source: Nat Aging. 2022 Oct 14;2(10):896–905. doi: 10.1038/s43587-022-00292-y (PMC10154205; doi:10.1038/s43587-022-00292-y)
Supplement: Supplementary file 1 — Supplementary Tables 1–3 [file 43587_2022_292_MOESM1_ESM.pdf]

nature aging

Letter

https://doi.org/10.1038/s43587-022-00292-y

Signs of immunosenescence correlate with poor outcome of mRNA COVID-19 vaccination in older adults

In the format provided by the authors and unedited

Extended Table 1: **Demographic characteristics of the study subjects.**

Parameter	Exposed (n = 49)			Vaccinated (n = 66)		
Female<br>Male	34 (69.4%)<br>15 (30.6%)			40 (60.6%)<br>26 (39.4%)		
Age (years)	54 (22-99)			54 (22-95)		
Days since positive PCR	79.0 (21-218)			NA		
Days since 1st vaccine shot	NA			44.0 (42-81)		
**Age Group (years)**	**Number of subjects**	**Median age**	**Gender**	**Number of subjects**	**Median age**	**Gender**
22 – 40	n = 15	32 (22 – 40)	M: 5 (33 %)<br>F: 10 (67 %)	n = 23	27 (22 – 40)	M: 14 (61 %)<br>F: 9 (39%)
41 – 65	n = 19	54 (41 – 61)	M: 6 (32 %)<br>F: 13 (68 %)	n = 25	54 (42 – 65)	M: 9 (36 %)<br>F: 16 (64 %)
≥ 66	n = 15	83 (75 – 99)	M: 4 (27 %)<br>F: 11 (73 %)	n = 18	84 (67 – 95)	M: 4 (22 %)<br>F: 14 (78 %)

Median (minimum-maximum); NA = not applicable

Extended Table 2: Full cohort description

	Vaccinated					
Subject Nr.	Gender	Age (years)	CharlsonIdx	Days post first BNT162b2 dose	Vaccination outcome	
1	Female	95	7	44	R	Older adults
2	Male	93	8	44	NR	
3	Female	93	8	44	R	
4	Female	93	7	42	R	
5	Female	88	6	44	R	
6	Female	88	6	42	NR	
7	Female	86	7	44	NR	
8	Female	86	6	44	R	
9	Female	84	8	44	NR	
10	Female	84	6	44	R	
11	Female	83	8	44	R	
12	Female	81	7	42	R	
13	Female	80	5	44	R	
14	Male	74	7	44	R	
15	Female	74	6	42	R	
16	Male	71	6	42	NR	
17	Male	70	4	42	R	
18	Female	67	4	42	R	
19	Male	65	2	42	R	Middle-aged
20	Female	61	2	42	R	
21	Male	60	3	67	R	
22	Female	59	2	42	NR	
23	Female	58	4	44	NR	
24	Female	58	1	60	R	
25	Male	58	3	42	R	
26	Female	56	1	55	R	
27	Female	56	1	42	R	
28	Female	56	1	55	R	
29	Female	55	3	42	R	
30	Female	55	1	47	R	
31	Male	54	1	65	R	
32	Female	54	1	81	R	
33	Male	54	1	60	R	
34	Male	54	1	44	R	
35	Female	53	2	42	R	
36	Female	51	1	62	R	
37	Male	47	0	60	R	
38	Female	45	2	44	R	
39	Male	45	0	42	R	
40	Female	44	0	49	R	
41	Female	44	0	42	R	
42	Female	44	0	44	R	
43	Male	42	0	42	R	
44	Female	40	0	42	R	Younger adults
45	Male	35	0	42	R	
46	Male	34	2	62	R	
47	Male	33	0	62	R	
48	Male	31	0	44	NR	
49	Male	31	0	67	R	
50	Female	29	0	60	**R**	
51	Male	29	0	60	R	
52	Female	29	0	63	R	
53	Male	28	0	51	R	
54	Male	28	0	67	R	
55	Female	27	1	64	R	
56	Male	26	0	60	R	
57	Male	26	0	60	R	
58	Female	26	0	60	R	
59	Male	26	0	47	R	
60	Female	26	0	63	R	
61	Male	25	0	58	R	
62	Female	25	0	60	R	
63	Female	25	0	56	R	
64	Male	23	0	62	R	
65	Female	22	0	74	R	
66	Male	22	0	58	R	

	Exposed				
Subject Nr.	Gender	Age (years)	CharlsonIdx	Days post PCR+	
1	Female	99	9	67	Older adults
2	Female	91	8	110	
3	Female	88	8	89	
4	Female	87	7	74	
5	Male	85	7	68	
6	Male	85	7	110	
7	Male	84	8	79	
8	Female	83	7	64	
9	Male	82	8	110	
10	Female	81	8	114	
11	Female	81	7	110	
12	Female	79	4	72	
13	Female	79	6	110	
14	Female	79	5	72	
15	Female	75	5	87	
16	Female	61	2	102	Middle-aged
17	Male	61	2	119	
18	Female	61	2	147	
19	Female	59	1	72	
20	Male	59	1	68	
21	Female	58	2	79	
22	Female	58	1	79	
23	Female	55	1	58	
24	Male	55	1	155	
25	Female	54	2	79	
26	Male	53	1	36	
27	Male	52	1	140	
28	Female	51	1	21	
29	Female	49	0	58	
30	Female	46	0	79	
31	Male	45	1	79	
32	Female	43	0	79	
33	Female	42	0	49	
34	Female	41	0	75	
35	Female	40	0	21	Younger adults
36	Female	39	0	NA	
37	Female	36	0	160	
38	Male	36	1	251	
39	Female	36	1	112	
40	Male	34	0	88	
41	Male	33	0	148	
42	Male	32	1	218	
43	Male	29	0	114	
44	Female	28	0	22	
45	Female	28	0	133	
46	Female	27	0	15	
47	Female	25	0	22	
48	Female	24	0	180	
49	Female	22	0	71	

R: Responder (>15 S-specific IFN-γ SFCs/10^6)

NR: Non-Responder (≤15 S-specific IFN-γ SFCs/10^6)

Extended Table 3. Antibodies used for FACS analyses

Antibody	Fluorochrome	Clone	Staining	Dilution	Company	Cat. number
PD-1	BV605	EH12.2H7	Surface	1:15	Biolegend	329924
CCR7	BV510	2-L1-A	Surface	1:40	BD	566760
CD45RA	PE-Cy7	HI100	Surface	1:120	BD	560675
CXCR5	PE	J252D4	Surface	1:10	Biolegend	356904
CD57	BB515	NK-1	Surface	1:300	BD	565945
CD14	APC-H7	MoP9	Surface	1:320	BD	560270
CD19	APC-H7	SJ25C1	Surface	1:80	BD	560252
KLRG-1	AF647	13F12F2	Surface	1:70	ThermoFisher	51-9488-42
CD3	APC-R700	UCH31	Intracellular	1:320	BD	565120
CD4	PerCP-Cy5.5	RTA-T4	Intracellular	1:40	Biolegend	300530
CD8	BV711	RTA-T8	Intracellular	1:120	BD	563677
IL-2	PE-CF594	5344.111	Intracellular	1:30	BD	562384
IFN-γ	BV421	B27	Intracellular	1:20	BD	562988
TNF-α	BV786	Mab11	Intracellular	1:10	Biolegend	502948
