# Supplementary material for: Signs of immunosenescence correlate with poor outcome of mRNA COVID-19 vaccination in older adults
Source: Nat Aging. 2022 Oct 14;2(10):896–905. doi: 10.1038/s43587-022-00292-y (PMC10154205; doi:10.1038/s43587-022-00292-y)
Supplement: Supplementary file 2 — Reporting Summary [file 43587_2022_292_MOESM2_ESM.pdf]

Corresponding author(s): G.F. Rimmelzwaan and G. Saletti

Last updated by author(s): Aug 15, 2022

Reporting Summary

Nature Portfolio wishes to improve the reproducibility of the work that we publish. This form provides structure for consistency and transparency in reporting. For further information on Nature Portfolio policies, see our Editorial Policies and the Editorial Policy Checklist.

Statistics

For all statistical analyses, confirm that the following items are present in the figure legend, table legend, main text, or Methods section.

n/a	Confirmed	
☐	☒	The exact sample size (*n*) for each experimental group/condition, given as a discrete number and unit of measurement
☐	☒	A statement on whether measurements were taken from distinct samples or whether the same sample was measured repeatedly
☐	☒	The statistical test(s) used AND whether they are one- or two-sided<br>*Only common tests should be described solely by name; describe more complex techniques in the Methods section.*
☐	☒	A description of all covariates tested
☐	☒	A description of any assumptions or corrections, such as tests of normality and adjustment for multiple comparisons
☐	☒	A full description of the statistical parameters including central tendency (e.g. means) or other basic estimates (e.g. regression coefficient) AND variation (e.g. standard deviation) or associated estimates of uncertainty (e.g. confidence intervals)
☐	☒	For null hypothesis testing, the test statistic (e.g. *F*, *t*, *r*) with confidence intervals, effect sizes, degrees of freedom and *P* value noted<br>*Give P values as exact values whenever suitable.*
☒	☐	For Bayesian analysis, information on the choice of priors and Markov chain Monte Carlo settings
☒	☐	For hierarchical and complex designs, identification of the appropriate level for tests and full reporting of outcomes
☐	☒	Estimates of effect sizes (e.g. Cohen's *d*, Pearson's *r*), indicating how they were calculated

Our web collection on statistics for biologists contains articles on many of the points above.

Software and code

Policy information about availability of computer code

Data collection	BDFACS Diva software v9.0 (BDBiosciences) was used to collect data from BD Fortessa X20 (BD Biosciences); ImmunoSpot Software (Version 7.0.20.0, ImmunoSpot, CTL) was used to collect data from the ImmunoSpot S6 Ultimate ELISpot reader.
Data analysis	FlowJo (Version 10.8.1; BD Biosciences); GraphPad Prism 9; ImmunoSpot Software (Version 7.0.20.0, ImmunoSpot); Microsoft Excel v.2016

For manuscripts utilizing custom algorithms or software that are central to the research but not yet described in published literature, software must be made available to editors and reviewers. We strongly encourage code deposition in a community repository (e.g. GitHub). See the Nature Portfolio guidelines for submitting code & software for further information.

Data

Policy information about availability of data

All manuscripts must include a data availability statement. This statement should provide the following information, where applicable:

- Accession codes, unique identifiers, or web links for publicly available datasets
- A description of any restrictions on data availability
- For clinical datasets or third party data, please ensure that the statement adheres to our policy

Raw FACS .fcs files have been uploaded in the ImmPort repository (www.https://www.immport.org/shared/home; access code SDY1961). Source data are provided with the paper

Field-specific reporting

Please select the one below that is the best fit for your research. If you are not sure, read the appropriate sections before making your selection.

☒ Life sciences ☐ Behavioural & social sciences ☐ Ecological, evolutionary & environmental sciences

For a reference copy of the document with all sections, see nature.com/documents/nr-reporting-summary-flat.pdf

Life sciences study design

All studies must disclose on these points even when the disclosure is negative.

Sample size	This is an observational study and samples size represents the number of samples available at the time of the analyses. Therefore, no prior sample size calculation was performed . However, a similar sample size has been used in other publications (Gao, L., Signal Transd. and target therapy 2021, https://doi.org/10.1038/s41392-021-00525-3; Zhang, W., Nat. Comm. 2022 https://doi.org/10.1038/s41467-022-30088-y; Roukens, A.H.E., Nature Imm. 2022, https://doi.org/10.1038/s41590-021-01095-w; Yang, X.L., Nature Comm. 2021, https://doi.org/10.1038/s41467-021-21155-x.
Data exclusions	No data were excluded
Replication	T cell measurements by FACS were performed once as limited number of PBMCs for some samples did not allow repetition. IFN-g ELISpot was carried out in triplicate wells or, in some cases (limited number of PBMCs), in duplicate and performed once.<br>SARS-CoV-2 antibody neutralization assay: sera were tested over a wide range of dilution in duplicate.
Randomization	This is an observational study.
Blinding	Donors received vaccination as part of routine health-care. SARS-CoV-2 convalescent subjects were selected from a larger prospective epidemiological study (Jonczyk R. Microbiol Spectr. 2022 Feb 23;10(1):e0151221. doi: 10.1128/spectrum.01512-21). Blinding was not relevant to our study because infectious status was laboratory confirmed and vaccination status known.

Reporting for specific materials, systems and methods

We require information from authors about some types of materials, experimental systems and methods used in many studies. Here, indicate whether each material, system or method listed is relevant to your study. If you are not sure if a list item applies to your research, read the appropriate section before selecting a response.

Materials & experimental systems

n/a	Involved in the study
☐	☒ Antibodies
☐	☒ Eukaryotic cell lines
☒	☐ Palaeontology and archaeology
☒	☐ Animals and other organisms
☐	☒ Human research participants
☒	☐ Clinical data
☒	☐ Dual use research of concern

Methods

n/a	Involved in the study
☒	☐ ChIP-seq
☐	☒ Flow cytometry
☒	☐ MRI-based neuroimaging

Antibodies

Antibodies used

FACS antibodies (stining)
mouse anti-human PD-1 (BV605), EH12.2H7, Surface 1:15, Biolegend, 329924
mouse anti-human CCR7 (BV510), 2-L1-A, Surface 1:40, BD, 566760
mouse anti-human CD45RA (PE-Cy7), HI100, Surface 1:120, BD, 560675
mouse anti-human CXCR5 (PE), J252D4, Surface 1:10, Biolegend, 356904
mouse anti-human CD57 (BB515), NK-1, Surface 1:300, BD, 565945
mouse anti-human CD14 (APC-H7), MoP9, Surface 1:320, BD, 560270
mouse anti-human CD19 (APC-H7), SJ25C1, Surface 1:80, BD, 560252
mouse anti-human KLRG-1 (AF647), 13F12F2, Surface 1:70, ThermoFisher, 51-9488-42
mouse anti-human CD3 (APC-R700), UCH31, Intracellular 1:320, BD, 565120
mouse anti-human CD4 (PerCP-Cy5.5), RTA-T4, Intracellular 1:40, Biolegend, 300530
mouse anti-human CD8 (BV711), RTA-T8, Intracellular 1:120, BD, 563677
mouse anti-human IL-2 (PE-CF594), 5344.111, Intracellular 1:30, BD, 562384
mouse anti-human IFN-γ (BV421), B27, Intracellular 1:20, BD, 562988
mouse anti-human TNF-α (BV786), Mab11, Intracellular 1:10, Biolegend, 502948

	Virus neutralization assay<br>Rabbit polyclonal anti- SARS-CoV-2 nucleocapsid, 1:1000, SinoBiological, 40588-T62<br>Anti-rabbit IgG Alexa Fluor 488, 1:1000, Thermo Fisher, A11008<br><br>Memory B cell ELISpot<br>Goat anti-human kappa antibody unlabeled, 6 µg/ml, Southern Biotech, 2060-01<br>Goat anti-human lambda antibody unlabeled, 6 µg/ml, Southern Biotech, 2070-01<br>Goat anti-human IgA- alkaline phosphatase detection antibody, 1:900, Southern Biotech, 2050-04<br>Goat anti-human IgG-horse radish peroxidase (HRP)detection antibody, 1:900, Southern Biotech, 2040-05<br><br>Purified antibodies<br>Mouse snti-Human CD3, 0.1 µg/ml, BD, 555336<br>Mouse anti-Human CD28/CD49d, 1 µg/ml , BD, 347690
Validation	All antibodies were commercially available. Antibody clones were selected based on validation data shown on the manufacturer's website. Specificity of the antibodies to human cells was reported by the manufacturers. Previous titration of antibodies were conducted on PBMCs to establish optimal antibody working dilutions. Spillover compensation was optimized suing compensation beads (BD ComBeads). Gating process was optimized using FMO controls.

Eukaryotic cell lines

Policy information about cell lines

Cell line source(s)	Vero cells from ATCC (CCL81)
Authentication	Cell lines were not authenticated
Mycoplasma contamination	Routinely tested and negative when used for neutralization assay.
Commonly misidentified lines<br>(See ICLAC register)	No commonly misidentified cell lines were used in this study.

Human research participants

Policy information about studies involving human research participants

Population characteristics	1) SARS-CoV-2 Exposed donors (n=49): aged 22-40 (5 male and 10 female), aged 41-65 (6 male and 13 female), ≥66 aged 75-99 (4 male and 11 female)<br>2) Vaccinated donors (n=66): aged 22-40 (14 male and 9 female), aged 41-65 (9 male and 16 female), ≥66 aged 67-95 (4 male and 14 female)
Recruitment	Recruitment of participants was performed as described in the open observational epidemiological study " Jonczyk R., et al., Combined Prospective Seroconversion and PCR Data of Selected Cohorts Indicate a High Rate of Subclinical SARS-CoV-2 Infections-an Open Observational Study in Lower Saxony, Germany, 2022, Microbiology spectrum doi: 10.1128/ spectrum.01512-21.." All subjects willing to participate were recruited for this study. We are unaware of any-self selection bias that might affect the results of the present study.<br>Study subjects belonged to three different groups located in the southern part of Lower Saxony, Germany: The first group, defined as "education/culture," included employees from universities, theater, schools, administration in and close to Hannover, Germany. The second group, defined as "company," consisted of employees of a biotechnological company in Goettingen, Germany. The third group included both employees and residents of retirement and nursing homes in and close to Hannover (designated as "nursing homes").
Ethics oversight	For these studies the Leibniz University (Hannover) obtained from the "Aerztekammer Niedersachsen" an ethical approval in August 2020 and amended in November 2021 (No. Bo/30/2010; Bo/31/2010; Bo/31/2010). A written informed consent was obtained from every subject, or legal representant.

Note that full information on the approval of the study protocol must also be provided in the manuscript.

Flow Cytometry

Plots

Confirm that:

- [ ] The axis labels state the marker and fluorochrome used (e.g. CD4-FITC).
- [ ] The axis scales are clearly visible. Include numbers along axes only for bottom left plot of group (a 'group' is an analysis of identical markers).
- [x] All plots are contour plots with outliers or pseudocolor plots.
- [x] A numerical value for number of cells or percentage (with statistics) is provided.

Methodology

Sample preparation

Blood samples for cellular analyses were collected into sodium heparin and EDTA tubes (Sarstedt), and Peripheral Blood Mononuclear Cells (PBMCs) isolated by density gradient centrifugation using Lymphoprep (Stemcell), following the manufacturer's protocol. Cells were frozen in freezing medium containing 90 % Fetal Bovine Serum (FBS, Thermo Fisher) and 10 % dimethyl sulfoxide (DMSO, Sigma Aldrich) and stored at -150 °C, until further use.
PBMCs were then thawed and a total of 2x10^6 of PBMCs were stimulated for 20 hours at 37 °C, 5% CO2 in a 96-well round-bottom plate using SARS-CoV-2 spike peptides pool (S1+S2; 0.5 μg/ml/peptide) As positive control, cells were stimulated with purified anti-CD3 (0.1 μg/ml; BD Biosciences), while equimolar amount of DMSO was used as negative control. Co-stimulatory anti-CD28 and anti-CD49d purified antibodies (1 μg/ml each; BD Biosciences) were added to all wells and Brefeldin A (7 μg/ml; Sigma Aldrich) included for the last 4 hours of incubation. Cells were first washed with Phosphate Buffered Saline (PBS), stained for 20 minutes at room temperature with LIVE/DEAD Fixable Near-IR stain kit (Molecular Probes) followed by 2 washes with PBS. After Fc receptor blocking for 20 minutes (Fc Block, BD Biosciences) antibodies for surface staining were added and incubated for 20 minutes. Next, cells were washed again, fixed/permeabilized using Cytofix/Cytoperm solutions (BD Biosciences) following the manufacturer's instruction. After two washes with PBS and Perm/Wash buffer (BD Biosciences), cells were blocked for 20 minutes with FcBlock and then antibodies diluted in Permwash buffer incubated for additional 20 minutes. Cells were washed twice and resuspended in PBS. All incubations were performed at room temperature in the dark. An average of 3x10^5 events per sample were acquired on a BD FortessaX20 (BD Biosciences).

Instrument

BD LSRFortessa™ X-20 Cell Analyzer (BD Biosciences)

Software

All data were analysed using FlowJo (Version 10.8.1; BD Biosciences)

Cell population abundance

Ex vivo analyses, no cell sorting was performed

Gating strategy

Cell population gating was based on the mean fluorescence intensity "minus one" (FMO) controls.

☒ Tick this box to confirm that a figure exemplifying the gating strategy is provided in the Supplementary Information.
